# Impact of operational conditions on methane yield and microbial community composition during biological methanation in in situ and hybrid reactor systems

**DOI:** 10.1186/s13068-021-02019-4

**Published:** 2021-08-21

**Authors:** Radziah Wahid, Svein Jarle Horn

**Affiliations:** Faculty of Chemistry, Biotechnology, and Food Science, Norwegian University of Life Sciences (NMBU), P.O. Box 5003, 1432 Ås, Norway

**Keywords:** Biological methanation, In situ, Hybrid, Hydrogenotrophic methanogens, CH_4_ yield

## Abstract

**Background:**

Biogas can be upgraded to methane biologically by adding H_2_ to biogas reactors. The process is called biological methanation (BM) and can be done in situ in a regular biogas reactor or the biogas can be transferred to a separate ex situ upgrading reactor. The hybrid BM concept, a combination of in situ and ex situ BM, has received little attention, and only a few studies have been reported. The hybrid BM has the advantage of resolving the issue of pH increment during in situ BM, while the size of the ex situ BM reactor could be reduced.

**Results:**

In this study, the efficiency of in situ and hybrid biological methanation (BM) for upgrading raw biogas was investigated. The hybrid BM system achieved a CH_4_ yield of 257 mL g_VS_^−1^ when degrading a feedstock blend of manure and cheese waste. This represented an increase in methane yield of 76% when compared to the control reactor with no H_2_ addition. A 2:1 H_2_:CO_2_ ratio resulted in stable reactor performance, while a 4:1 ratio resulted in a high accumulation of volatile fatty acids. H_2_ consumption rate was improved when a low manure–cheese waste ratio (90%:10%) was applied. Furthermore, feeding less frequently (every 48 h) resulted in a higher CH_4_ production from CO_2_ and H_2_. *Methanothermobacter* was found to dominate the archaeal community in the in situ BM reactor, and its relative abundance increased over the experimental time. *Methanosarcina* abundance was negatively affected by H_2_ addition and was nearly non-existent at the end of the experiment.

**Conclusions:**

Our results show that hybrid BM outperforms in situ BM in terms of total CH_4_ production and content of CH_4_ in the biogas. In comparison to in situ BM, the use of hybrid BM increased CH_4_ yield by up to 42%. Furthermore, addition of H_2_ at 2:1 H_2_:CO_2_ ratio in in situ BM resulted in stable reactor operation.

## Background

Renewable electricity from photovoltaics and wind turbines could play a significant role in the future European electricity system [1]. However, wind and solar are intermittent energy sources, necessitating long-term and large-scale storage capacity in order to store renewable electricity during excess and supply electricity during shortage [2]. One solution is to store electricity in batteries, but it has disadvantages, including high cost of manufacture, low storage capacity and use of rare minerals [3]. Another storage alternative is to use excess electricity from wind or solar energy to generate H_2_ via water electrolysis [4]. However, the use of H_2_ as a renewable energy carrier presents significant challenges that have not yet been addressed, linked to its low density requiring a high storage capacity infrastructure, while the direct use of H_2_ as transport fuel is still under development [5, 6]. However, H_2_ may be combined with CO_2_ produced in existing biogas plants and converted to CH_4_, for which large-scale infrastructure and applications are in place [4]. This concept of converting electrical into chemical energy is known as power-to-methane (PtM) [7].

PtM can be achieved in two ways, either by thermochemical methanation (TM) or BM [1]. Both methods are based on the Sabatier reaction (Eq. 1), in which four moles of H_2_ react with one mole of CO_2_ to produce one mole of CH_4_ and two moles of H_2_O [8]:

1 $$4{\text{H}}_{2} + {\text{CO}}_{2} \to {\text{CH}}_{4} + 2{\text{H}}_{2} {\text{O}}\,\,\,\Delta {\text{G}}^{{0^{\prime } }} = - 130{\text{ kJ mol}}^{ - 1}$$

In comparison to other biogas upgrading technologies (water scrubbing, pressure swing adsorption, and membrane separation), the methanation approach minimizes CO_2_ (in biogas) losses to the environment as CO_2_ is converted into CH_4_ during the process [9].

Metal catalysts such as Ni and Al_2_O_3_ are used in TM, which operates at high temperatures (between 200 and 500 °C) and pressures (up to 100 bar). The metal catalyst is sensitive to contaminants such as hydrogen sulphide (H_2_S), so high purity of the reactant gases is required [2]. BM, on the other hand, uses a biological catalyst (methanogenic archaea) and operates at mild temperatures (35–65 °C) and pressures (< 15 bar). In addition, as opposed to TM, the process tolerates impurities such as H_2_S [2]. At present, BM is gaining more attention as a result of its advantages, and a growing number of studies have been dedicated to it [10–12]. Previous research has reported three types of BM concepts: in situ [12, 13], ex situ [14, 15], and hybrid [16].

In situ BM is attractive since biogas is upgraded directly in the biogas reactor without incurring additional costs for a secondary reactor. However, some technical challenges have been reported in previous studies [12, 13] such as increased pH (> 8.5) due to bicarbonate removal to CH_4_ and high H_2_ partial pressure (exogenous H_2_), which inhibits the activity of specific bacteria and methanogens. Furthermore, the low H_2_ gas–liquid mass transfer rate limits methanogen uptake of H_2_ for CO_2_ to CH_4_ conversion, which is a key challenge for both in situ and ex situ BM [17]. Ex situ BM involves the injection of CO_2_ from biogas (or other sources) and H_2_ into a separate reactor containing hydrogenotrophic methanogens (pure or enriched culture) for CH_4_ conversion [6]. The hybrid BM concept (combination of in situ and ex situ), on the other hand, has received little attention, and only a few studies have been conducted. In the hybrid system, H_2_ is added to the main biogas reactor for in situ upgrading of CO_2_ to CH_4_ and the produced biogas (including residual H_2_) is transferred to an upgrading ex situ reactor for further CH_4_ production. The hybrid BM has the advantage of addressing the issue of pH increment during in situ BM, while a smaller reactor can be used for ex situ BM [6]. Furthermore, the hybrid system incorporates in situ and ex situ configurations, implying that the BM process occurs twice, increasing the residence time of H_2_ in the system. Corbellini et al. [16] used a two-stage thermophilic reactor to investigate the performance of hybrid BM and obtained final CH_4_ concentrations of more than 95% in some experiments. The hybrid concept was also proposed by Voelklein et al. [18] for full-scale application as an alternative to conventional upgrading systems.

The goal of this study was to assess the performance of a hybrid BM system in terms of substrate conversion efficiency and biogas quality using a 10-L continuous-stirred tank reactor (CSTR) (in situ) and a 2-L reactor with packing materials (ex situ). A similar 10-L CSTR reactor without H_2_ addition was used as a control. Furthermore, the performance of in situ and hybrid systems was compared in order to evaluate the capability of hybrid BM in resolving technical challenges associated with in situ, such as pH increment and low H_2_ gas–liquid mass-transfer rate. This work also investigated parameters (e.g., H_2_:CO_2_ ratio, stirring speed, and feeding frequency) that affect the efficiency of in situ BM and the composition and dynamics of the microbial populations. Parameters such as pH, total ammonium nitrogen (TAN), volatile fatty acids (VFA), and methane yield and content were closely monitored during the experiment.

## Results and discussion

### Process performance and biogas upgrading of in situ BM

**Fig. 1:**
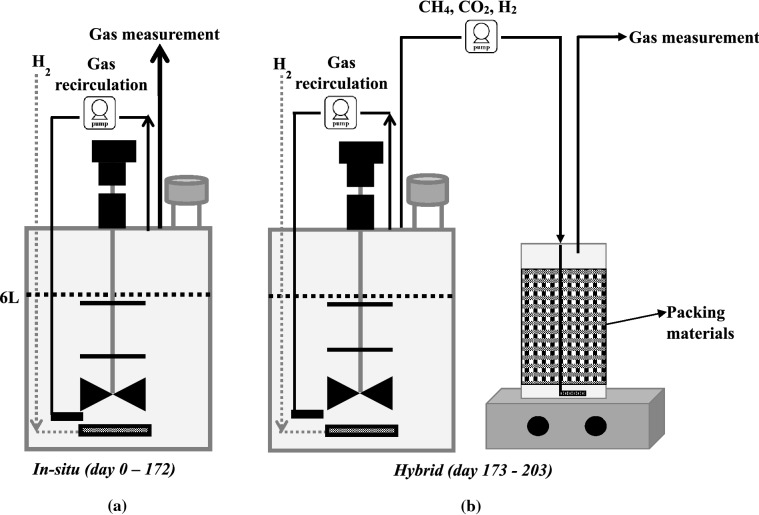
**a** Illustration of in situ and **b** hybrid reactor setups

**Fig. 2:**
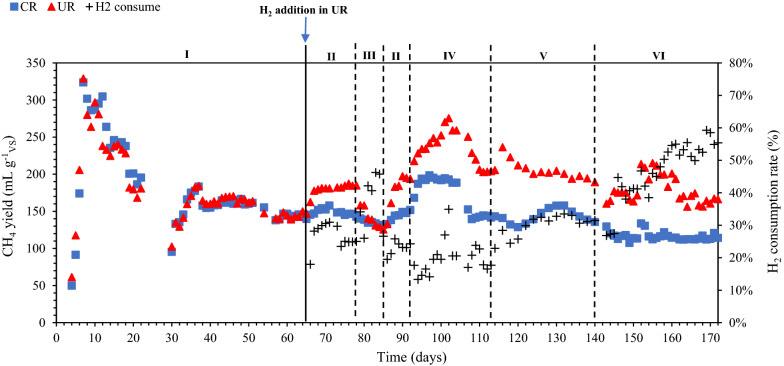
Methane yield and H_2_ consumption at different experimental phases (I–VI). *CR* control reactor; *UR* in situ upgrading reactor; *H*_*2*_* consumed* H_2_ consumption

**Fig. 3:**
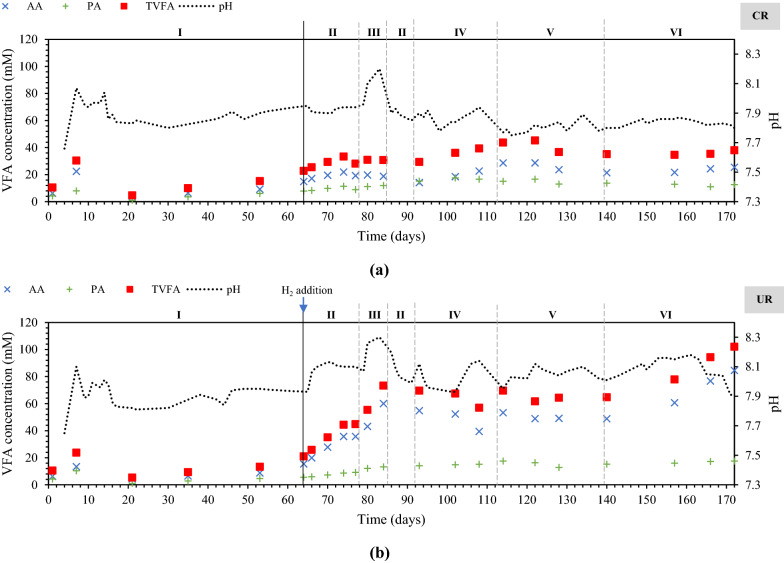
pH and volatile fatty acid concentrations in **a** control reactor (CR) and **b** in situ upgrading reactor (UR). *AA* acetic acid; *PA* propionic acid; *TVFA* total volatile fatty acids

Figure 1 illustrates the in situ and hybrid reactor configurations. The characteristics of the inoculum and the applied substrates are given in Table 1. Operating parameters and performance data for the 10-L control and upgrading reactors (CR, UR) under steady-state conditions are summarized in Tables 2 and 3, respectively. The experiment was conducted for 172 days and divided into six phases. Figures 2 and 3 illustrate the changes in methane yield, pH, and VFAs over the experimental period for upgrading and control reactors.

**Table 1:** Characteristics of inoculum and substrates

	TS (%)	VS (%)	pH	TAN (g L^−1^)	TVFA (mM)
Inoculum	3.04	1.83	8.07 ± 0.01	1.54	7.83 ± 2.13
Cow manure	9.35 ± 0.25	7.66	7.34 ± 0.02	1.24 ± 0.20	64.06 ± 0.94
Cheese waste	12.64	11.64	4.78 ± 0.01	0.14	7.58 ± 1.17
Feed (cow manure + cheese waste)	9.92	8.26 ± 0.01	7.05 ± 0.01	1.20 ± 0.12	60.09 ± 2.95

*TS* total solid; *VS* volatile solid; *TAN* total ammonium nitrogen; *TVFA* total volatile fatty acids

**Table 2:** Operating conditions of control- and in situ upgrading reactors at different experimental phases

Parameters	Unit	Phases											
		I (day 1–64)		II (day 65–78)		III (day 79–85)		IV (day 93–113)		V (day 114–140)		VI (day 141–172)	
		CR	UR	CR	UR	CR	UR	CR	UR	CR	UR	CR	UR
Stirring speed	rpm	80	80	80	80	140	140	80	80	80	80	80	80
CM:CW ratio	%	10	10	10	10	10	10	20	20	10	10	10	10
Feeding frequency	hours	24	24	24	24	24	24	24	24	48	48	24	24
H_2_:CO_2_ ratio	–	–	–	–	2	–	2	–	2	–	2	–	4

Day 86–92—same conditions as phase II

*CR* control reactor; *UR* in situ upgrading reactor; *CM* cow manure; *CW* cheese waste

**Table 3:** Performance data for control and upgrading reactors at different experimental phases (mean  ±  SD)

Phases	I		II		III		IV		V		VI	
Reactor	CR	UR	CR	UR	CR	UR	CR	UR	CR	UR	CR	UR
Biogas yield (mL g^−1^_VS_)	244.72 ± 8.29	241.15 ± 11.01	245.59 ± 4.52	298.11 ± 4.84	232.47 ± 4.16	218.43 ± 9.70	263.18 ± 7.16	349.90 ± 3.81	231.00 ± 4.49	305.43 ± 2.55	205.68 ± 6.18	246.00 ± 4.65
CH_4_ yield (mL g^−1^_VS_)	144.77 ± 2.38	143.50 ± 3.95	146.34 ± 2.25	185.44 ± 1.94	133.52 ± 2.22	132.96 ± 5.20	142.19 ± 1.83	204.15 ± 1.48	141.70 ± 6.05	193.79 ± 4.24	134.03 ± 2.58	164.60 ± 2.95
Gas compositions (%)												
CH_4_	58.24 ± 1.09	59.14 ± 1.25	59.88 ± 0.66	39.97 ± 0.60	57.57 ± 0.13	40.76 ± 0.45	53.70 ± 0.36	38.69 ± 0.38	58.55 ± 0.50	42.58 ± 0.59	56.10 ± 1.55	38.65 ± 0.77
CO_2_	41.76 ± 1.09	40.86 ± 1.25	40.41 ± 0.68	28.59 ± 0.59	42.43 ± 0.13	26.19 ± 0.14	46.30 ± 0.36	28.04 ± 0.44	41.45 ± 0.50	23.13 ± 0.51	43.90 ± 1.55	19.11 ± 0.24
H_2_	–	–	–	31.44 ± 0.15	–	33.05 ± 0.31	–	33.27 ± 0.11	–	34.29 ± 0.08	–	42.24 ± 0.83
H_2_ consumption (%)	–	–	–	24.96 ± 0.09	–	45.99 ± 0.36	–	17.35 ± 0.70	–	31.80 ± 1.12	–	53.85 ± 2.71
pH	7.92 ± 0.02	7.94 ± 0.01	7.94 ± 0.01	8.10 ± 0.01	8.15 ± 0.07	8.28 ± 0.03	7.91 ± 0.03	8.11 ± 0.03	7.82 ± 0.06	8.04 ± 0.05	7.77 ± 0.02	7.95 ± 0.08
TVFA (mM)	18.99 ± 5.33	17.12 ± 5.37	30.73 ± 3.68	44.55 ± 0.36	30.56 ± 0.45	66.63 ± 9.85	37.67 ± 2.29	62.18 ± 7.42	40.18 ± 5.06	65.08 ± 3.28	36.66 ± 1.89	98.14 ± 5.50
AA (mM)	12.04 ± 4.02	12.07 ± 4.71	20.58 ± 1.91	35.68	18.32 ± 1.68	53.50 ± 9.05	20.57 ± 2.97	45.83 ± 9.10	25.59 ± 3.66	50.01 ± 2.19	24.87 ± 0.79	80.63 ± 5.54
PA (mM)	6.95 ± 1.31	5.05 ± 0.66	10.15 ± 1.77	8.87 ± 0.36	12.24 ± 1.23	13.13 ± 0.95	17.10 ± 0.67	16.36 ± 1.67	14.58 ± 1.59	15.07 ± 1.56	11.79 ± 1.10	17.51 ± 0.04
TAN (g L^−1^)	2.48 ± 0.06	2.52 ± 0.02	2.57 ± 0.01	2.77 ± 0.16	3.32 ± 0.22	2.88 ± 0.14	3.12 ± 0.11	3.17 ± 0.03	2.80 ± 0.15	2.89 ± 0.09	2.65 ± 0.11	2.80 ± 0.07

*CR* control reactor; *UR* in situ upgrading reactor; *TVFA* total volatile fatty acid; *AA* acetic acid; *PA* propionic acid

### ***Phase I: initial phase—without H***_***2***_*** addition***

In this phase, the two reactors (CR and UR) were operated identically and showed very similar performance in terms of biogas production (241–245 mL g^−1^_VS_) and CH_4_ yield (144–145 mL g^−1^_VS_) (Table 3). The average CH_4_ content of the reactors (58–59%) and the pH (7.9) were also similar. The total VFA content was around 18 mM, with acetic acid (AA) accounting for more than 60% of the total VFAs. The ratio of propionic acid (PA) to AA of both reactors was below 1.4, indicating a stable AD process according to [19]. The TAN concentration was around 2.5 g L^−1^. The values align well with those obtained by [20], who observed that a TAN value of 2.5 g L^−1^ (pH 7.9) resulted in stable biogas production during thermophilic (55 °C) anaerobic digestion of cow manure.

### ***Phase II: initial H***_***2***_*** phase***

H_2_ was added in UR from day 64 at a flow rate of 3 mL min^−1^, corresponding to a H_2_:CO_2_ ratio of 2:1. As shown in Fig. 2, CH_4_ yield increased immediately after H_2_ addition and stabilized from day 70. The average CH_4_ yield of UR was 185 mL g^−1^_VS_, which was approximately 27% higher than the average CH_4_ yield of CR (Table 3). A similar observation was reported by Treu et al. [21] where H_2_ addition into a CSTR at a 2:1 ratio resulted 13% increase in CH_4_ yield. The pH of UR increased from 7.94 to 8.10, while the pH of CR remained the same as in phase 1. BM resulted in a rise in pH due to the removal of CO_2_ from the liquid phase. Bicarbonate ions (HCO_3_^−^) are produced during the AD process when CO_2_ reacts with OH in the liquid phase, contributing to the buffering capacity of the reactor. Addition of H_2_ to the system resulted in CO_2_ consumption and thus loss of buffering capacity [15]. Similar findings have been reported in previous studies [12, 21, 22]. Total VFA levels in UR rose to more than double the amount in phase I. In contrast to our study, Treu et al. [21] reported relatively low and stable VFA levels after H_2_ addition.

In CR, the average AA concentration was 21 mM, while in UR, it was 36 mM. PA levels were slightly higher in both reactors than in phase 1. TAN concentrations were also elevated, with 2.57 g L^−1^ for CR and 2.77 g L^−1^ for UR. The H_2_ consumption rate of UR was calculated to be 25%, corresponding to a CH_4_ production rate of 0.04 mL L^−1^ d^−1^.

### Phase III: increased stirring speed

In phase III, the stirring speed of both reactors was increased from 80 to 140 rpm (day 79) in an attempt to improve the transfer of H_2_ to the liquid phase in UR. As shown in Fig. 2, the CH_4_ yield from UR decreased significantly as the stirring speed increased. The CH_4_ yield of UR was reduced from 185 (day 78) to 126 mL g^−1^_VS_ (day 85) for UR. The decrease in CH_4_ yield of UR was corroborated by the accumulation of acetate (67 mM on average), which was nearly double of what was measured in phase II (Fig. 3b). Besides, the propionate concentration was slightly increased from 9 to 13 mM. These observations could indicate that parts of the microbial community were negatively affected by the higher share forces at 140 rpm. Vavilin et al. [23] reported that high-intensity mixing inhibits methanogenesis and hydrolysis/acidogenesis, and that the anaerobic digestion outcome is dependent on the concentration of methanogenic biomass. Furthermore, Sindall et al. [24] found that increased stirring speed (200 rpm) disturbs localized pockets of acetate, resulting in a decrease in the ratio of acetoclastic methanogens to hydrogenotrophic methanogens.

Regardless of the fact that the total CH_4_ yield decreased as the stirring speed increased, the H_2_ consumption rate in UR increased from 25 to 46%. This observation was in agreement with our previous study [25]. The rate of CH_4_ production from H_2_ and CO_2_ conversion was increased from 0.04 to 0.08 mL L^−1^ d^−1^. For the CR, the CH_4_ yield was reduced from 143 to 131 mL g^−1^_VS_. Ghanimeh et al. [26] observed a decrease in CH_4_ yield when stirring speed was increased from 80 to 120 rpm. No AA accumulation was observed in the CR, whereas the PA level was slightly higher than in phase II (12 mM) (Fig. 3a; Table 3). The pH in both reactors was higher than in phase II, with pH of 8.15 and 8.28 for CR and UR, respectively. The elevated pH in UR can be attributed to greater CO_2_ consumption in the liquid as a result of the increased H_2_ gas–liquid mass transfer rate at higher stirring speeds and thus higher BM activity [1].

### Phase IV: change of feedstock blend ratio

On day 86, the stirring speed was again reduced to 80 rpm (return to Phase II conditions), and the CH_4_ yield rose significantly until it reached a plateau from day 90 (Fig. 2). From day 92 the CW fraction was increased from 10 to 20% on day 93 (Phase IV), resulting in an OLR of 0.78 g_VS_ L^−1^ d^−1^. The CH_4_ yield increased in both reactors, with maximum values being 195 mL g^−1^_VS_ (CR) and 276 mL g^−1^_VS_ (UR) (Fig. 2). After day 102, however, the CH_4_ yield gradually decreased until it reached a stable period around day 111. During the stable period, the average CH_4_ yields of CR and UR were 142 mL g^−1^_VS_ and 204 mL g^−1^_VS_, respectively (Table 3). The average CH_4_ yield of CR measured in this study was lower than that measured by Comino et al. [27] (similar feedstock blend, 80% CM: 20% whey), despite the fact that both studies had comparable CH_4_ content (53%). Longer HRT (41 days) and higher OLR (3.33 g_VS_ L^−1^d^−1^) were used by Comino et al. which may explain the difference in performance. The average CH_4_ content of UR was 39%. The H_2_ consumption rate was around 17%, which was 31% lower than the consumption rate when CW fraction was set at 10%. The total VFA content of CR was slightly higher towards the end of phase IV (Fig. 3a), while the total VFA content of UR was relatively stable (Fig. 3b). The pH of both reactors was lower than in phase III, with an average pH of 7.91 for CR and 8.11 for UR. Increased CW ratio to 20% resulted in higher TAN values (both reactors) compared to phase II, suggesting more thorough CW degradation as TAN is a product of protein degradation.

### Phase V: feeding frequency

In phase V, the CW fraction was reduced to 10% and the substrate feeding frequency was changed to once every 48 h (instead of once per 24 h). In terms of CH_4_ yield for CR, no changes were observed, while CH_4_ yield for UR was gradually reduced until a stable period was achieved (day 134). The average CH_4_ yield for CR was 139 mL g^−1^_VS_ and 194 mL g^−1^_VS_ for UR. The CH_4_ yield of UR in phase IV was slightly higher than in phase II (feeding every 24 h). The H_2_ consumption rate was higher than phase II (24 h feeding) when the reactor was fed every 48 h (25% vs 32%). The increased CH_4_ yield and H_2_ consumption rate in UR could be attributed to enrichment of hydrogenotrophic methanogens in less frequent feeding. According to Piao et al. [28], reducing substrate feeding frequency tended to increase the abundance of H_2_-utilizing methanogens. When substrate feeding frequency was reduced from every 24 h to every 48 h, the abundance of hydrogenotrophic methanogens increased from 45 to 53% [28]. The average total VFA content for CR and UR were 26 and 50 mM, respectively. The pH of both reactors was slightly lower than in phase II.

### Phase VI: increased H_2_:CO_2_ ratio

Substrate feeding was changed to once daily starting on day 141, and the H_2_ flow rate was increased to 6 mL min^−1^, equivalent to a 4:1 H_2_:CO_2_ ratio (Phase VI). The increased H_2_:CO_2_ ratio initially boosted CH_4_ yield in UR with a maximum at day 151. However, the yield fell after day 163. The average CH_4_ yield in this period was 165 mL g^−1^_VS_, about 11% lower than the value in phase II (H_2_:CO_2_ ratio  =  2:1). Despite the lower CH_4_ yield, the H_2_ consumption rate was doubled (54%) compared to phase II (25%) due to the increased H_2_:CO_2_ ratio, which probably stimulated H_2_-consuming anaerobic microbes.

AA accumulated toward the end of the phase, reaching a maximum concentration of 85 mM. The increase in AA levels may be explained by the inhibition of acetoclastic methanogens (e.g., *Methanosarcina*) caused by high H_2_ partial pressure [29] or by the enrichment of particular microbial pathways such as homoacetogenesis (Wood–Ljungdahl pathway) [6]. PA content was also increased from 15 to 18 mM when the H_2_:CO_2_ ratio was increased. The rise in total VFA content coincided with a drop in pH from 8.01 to 7.91. For CR, the CH_4_ yield remained consistent throughout phase VI, with an average of 134 mL g^−1^_VS_. The average total VFA concentration was 21 mM, with a pH of 7.82. AA concentration accounted for 58% of the total VFA content. The TAN concentration was 2.65 g L-1, which was similar to the value observed in phase II (2.57 g L^−1^).

### In situ vs. hybrid configurations

A hybrid configuration was tested at the end of the experiment (after day 172). An additional 2-L reactor filled with packing materials was used as an ex situ biogas upgrading reactor (HR) for the biogas from UR (Fig. 1b). Initially, the operating parameters of UR were adjusted to the same as in phase II with a H_2_:CO_2_ ratio of 2:1. The gas yield from hybrid configurations (Table 4) represent the gas yield from both in situ and ex situ reactors.

**Table 4:** Performance of hybrid reactor system at different H_2_:CO_2_ ratios (mean  ±  SD)

H_2_:CO_2_ ratio	pH^a^	TAN^a^ (g L^−1^)	AA^a^ (mM)	CH_4_ yield^b^ (mL gVS^−1^)	H_2_ consumptions^b^ (%)	CH_4_ content (without considering H_2_)^b^ (%)	Output gas compositions^b^ (%)		
							CH_4_	CO_2_	H_2_
2:1	8.07	1.09	4.12	257.27 ± 4.28	60.23 ± 0.75	79.89 ± 1.40	63.20 ± 1.44	16.10 ± 1.18	20.70 ± 0.43
4:1	8.06	1.01	4.23	234.15 ± 3.70	62.22 ± 2.63	73.09 ± 2.22	50.58 ± 0.93	18.64 ± 1.75	30.78 ± 0.83

CH_4_ content (without considering H_2_)  =  %CH_4_/(%CH_4_  +  %CO_2_  ×  100)

The CH_4_ yield and content, as well as the output gas compositions of hybrid BM, represent the total outcome of both in situ and ex situ reactors

*TAN* total ammonium nitrogen; *AA* acetic acid

^a^Parameters measured in ex situ upgrading reactor (HR)

^b^Data from hybrid system [in situ (UR)  +  ex situ (HR)]

**Fig. 4:**
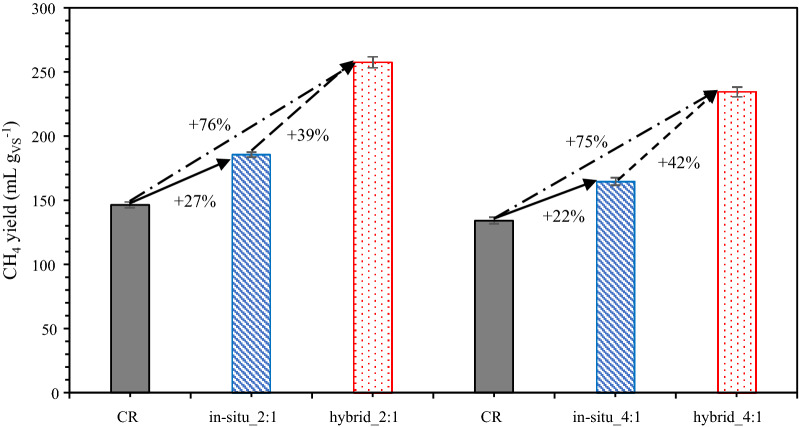
Average methane yield of control reactor (CR), in situ upgrading reactor (UR) and hybrid upgrading reactor (HR). *2:1—H*_*2*_ CO_2_ ratio of 2:1, 4:1—H_2_:CO_2_ ratio of 4:1

When the hybrid setup was used instead of an in situ (phase II), 39% extra CH_4_ was obtained (Fig. 4). The average CH_4_ yield rose from 185 to 257 mL g^−1^_VS_. Furthermore, the H_2_ consumption rate increased by twofold compared to in situ (phase II), and the average CH_4_ content increased from 40 to 63% (Tables 3, 4). The CH_4_ content without considering H_2_ from hybrid system was around 80%. When compared to the control reactor (Fig. 4), the hybrid configuration resulted in a 76% higher CH_4_ yield, while in situ configuration resulted in 27% more CH_4_ (Fig. 4). HR had an average pH of 8.07 and an AA concentration of approximately 4.12 mM. The TAN concentration of HR was around 1.09 g L^−1^.

The H_2_:CO_2_ ratio was increased to 4:1 after a stable condition was observed. The average CH_4_ yield fell from 257 to 234 mL g^−1^_VS_ (approximately 9% less CH_4_). The average CH_4_ content was reduced from 63 to 51%. Nonetheless, the H_2_ consumption rate (62%) was slightly higher than at the 2:1 H_2_:CO_2_ ratio (60%), indicating that acetate-oxidizing bacteria had the capacity to consume more H_2_ to produce acetate, as observed in phase VI. Compared to in situ configuration (phase VI), about 42% extra CH_4_ was measured and approximately 75% more CH_4_ was produced when compared to control (Fig. 4). The concentrations of AA and TAN were equivalent to those found at a 2:1 H_2_:CO_2_ ratio.

Compared to Corbellini et al. [16] our study resulted in lower upgraded CH_4_ content of in situ BM. This may be attributed to differences in reactor working volume, as a larger working volume (6 L) was used in the present study compared to 3 L in [16]. Our findings were more comparable to those of [18], who used a 9-L working volume for in situ testing. Furthermore, when a 4:1 H_2_:CO_2_ ratio was added to UR in our study, AA accumulation (> 4 g L^−1^) was observed, leading to a decrease in pH, while VFA level observed in [16] was maintained at 2 g L^−1.^

To prevent process instability in in situ BM reactor, we propose that the amount of H_2_ added to the in situ reactor should be kept at a relatively low H_2_:CO_2_ ratio (e.g., 2:1). This will minimize the increase in pH caused by bicarbonate removal as well as the possible inhibition of some anaerobic bacteria that are sensitive to high H_2_ partial pressure. Our study discovered residual H_2_ in the in situ and hybrid BM reactors, indicating that further optimization is required. A pressurized reactor may be a solution. Increased operating pressure enhances the solubility of gases and decreases bubble size. Smaller bubble size is beneficial since it maximizes the contact area between bacteria and gaseous substrates while slowing gas upflow through the reactor [1, 30]. Previous research found that increasing reactor pressure during in situ and ex situ BM resulted in improved conversion efficiency [31, 32]. A very high CH_4_ concentration (> 98%) in the biogas was reported when reactor pressure was set between 5 and 15 bars for a 5 m^3^ ex situ CSTR [33]. Additionally, the design of the ex situ reactor used in our study can be improved, for example, by using a long column design like trickle-bed reactor.

### Microbial community composition

**Fig. 5:**
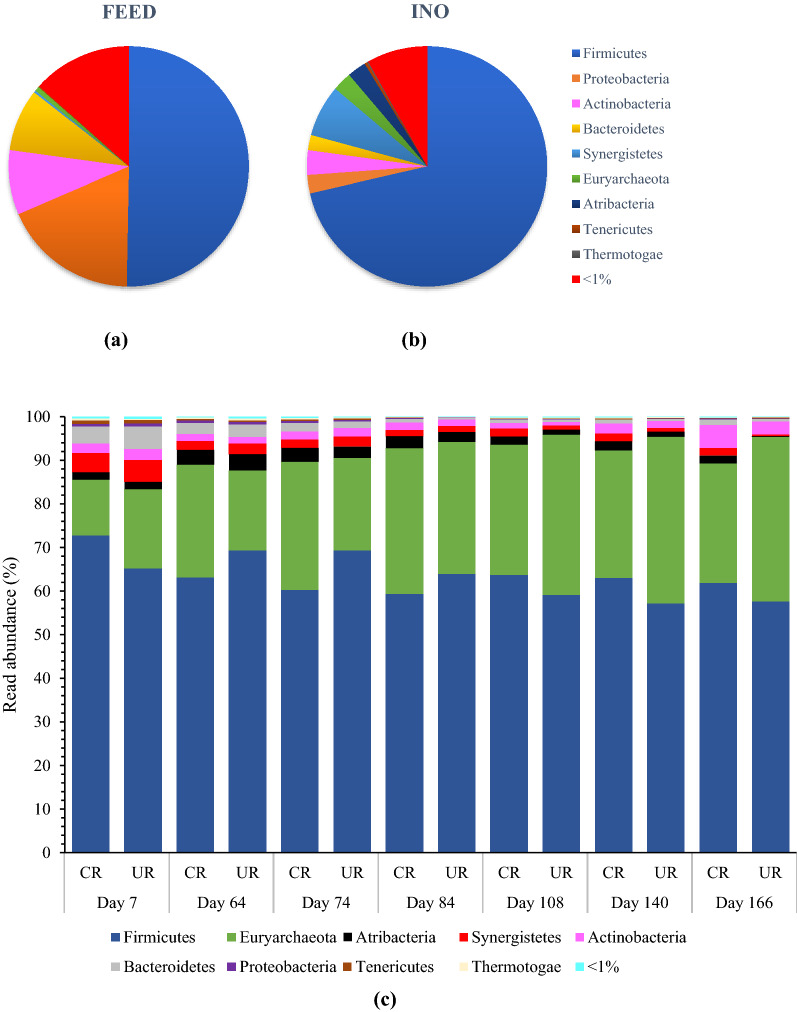
Stacked bar plot of the read abundance (%) of phyla detected in **a** feed, **b** inoculum and **c** control reactor (CR) and in situ upgrading reactor (UR) over time. Phyla are indicated by the colours displayed in the legend of the figure. Samples with less than 1000 sequences were omitted from the figure. FEED is the biogas substrate used in the study (90%CM:10%CW) and INO indicates the inoculum

Microbial analysis of the reactor feed (90% CM: 10% CW) showed that *Firmicutes* and *Proteobacteria* were the two dominant bacterial phyla, accounting for approximately 50 and 18% of the abundance, respectively (Fig. 5a). Other phyla present in the feed included *Actinobacteria* (9%) and *Bacteroidetes* (8%). Analysis of the inoculum microbiology showed that *Firmicutes* was the dominating phylum (71%), followed by *Synergistetes* (7%), *Actinobacteria*, and *Euryarchaeota* (both phyla accounted 3% abundance) (Fig. 5b). *Atribacteria* and *Thermotogae* were also detected in the inoculum, but they were not found in the feed sample.

The taxonomic classification of the microbial community revealed that *Firmicutes* were the most abundant phyla n the reactors, accounting for 57–72% of relative abundance depending on the time points (Fig. 5c). This is in agreement with the findings of [34] where *Firmicutes* dominated a thermophilic biogas reactor digesting cow manure. *Firmicutes* engages in a variety of metabolic processes for carbohydrate and fatty acid degradation, including the Wood–Ljungdahl pathway (homoacetogenesis) and syntrophic acetate oxidation, which explains their abundance in the reactors [12]. *Clostridia*, which belong to the *Firmicutes*, was the most abundant class (representing more than 33% of all bacterial sequences). Other bacterial phyla, such as *Synergistetes* and *Bacteroidetes,* were present in both reactors at first, but their numbers declined over time. In terms of methanogenic population, the abundance of *Euryarchaeota* varied over time, between 13 and 33% for CR, and 18–38% for UR (Fig. 5c).

**Fig. 6:**
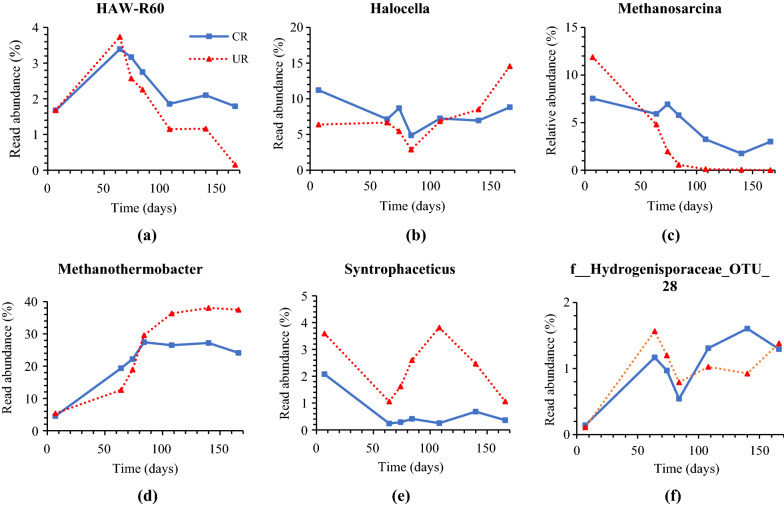
Genus abundance (%) of **a** *HAW-R60*, **b** *Halocella*, **c** *Methanosarcina*, **d** *Methanothermobacter*, **e** *Syntrophaceticus*, and **f** *f_Hydrogenisporaceae_OTU_28* in the control and in situ upgrading reactors over time. Samples with less than 1000 sequences were omitted from the figure. *CR* control reactor; *UR* in situ upgrading reactor

Some bacteria, such as *HAW-R60*, an *Atribacteria* phyla, was clearly negatively affected by H_2_ addition (Fig. 6a). Their abundance declined over time and was nearly non-existent in phase VI*. Atribacteria* have been found previously in thermophilic biogas reactors and are involved in hydrolysis of polysaccharides [35]. Another hydrolytic bacterium, *Halocella*, behaved differently, reaching highest abundance when the H_2_:CO_2_ ratio was increased to 4:1 (phase VI) (Fig. 6b). Their abundance in UR increased from 6.7 (without H_2_ addition) to 14.6%. The increase in stirring speed in phase II (day 79–85) seemed to negatively affect *Halocella*, with decreased abundance in both CR and UR. The cellulolytic bacteria *Halocella* belong to the class *Clostridia* and is responsible for cellulose degradation and produces ethanol and H_2_ from lignocellulosic substrates [36]. In addition, it has been reported that *Halocella* have enzymes for hemicellulose and starch degradation [37]. *Halocella* have mainly been found in manure-based samples and their presence in thermophilic biogas reactor has been reported previously [38].

Within the domain archaea, *Methanosarcina* was the only detected methanogen capable of acetoclastic methanogenesis, although it can also carry out hydrogenotrophic methanogenesis [39]. *Methanosarcina* was clearly negatively affected by H_2_ addition and disappeared from UR after 108 days (Fig. 6c). High H_2_ partial pressure has previously been shown to be detrimental to *Methanosarcina* [40]. Furthermore, the observed accumulation of AA in UR (Fig. 3b) is consistent with *Methanosarcina* inhibition. The abundance of *Methanosarcina* in UR decreased even more when the stirring speed was increased to 140 rpm (phase III). This observation can be explained by an increase in dissolved H_2_ in the reactor, which also corresponded with an increase in H_2_ consumption (Table 3).

In contrast to *Methanosarcina*, the hydrogenotrophic methanogen *Methanothermobacter* increased in abundance over time and responded positively to H_2_ addition. *Methanothermobacter* are typical hydrogenotrophic methanogens that are commonly found in thermophilic biogas reactors [41]. As shown in Fig. 6d, their abundance in UR got higher than the abundance in CR over time, suggesting that they were enriched as a result of H_2_ addition. The high abundance of *Methanothermobacter* found in this study is consistent with previous research that found this genus to be dominant in thermophilic biogas upgrading systems [6, 15, 42]. According to [43], *Methanothermobacter* expand rapidly when H_2_ is abundant and are adaptable to different concentrations of dissolved H_2_.

*Syntrophaceticus* abundance increased rapidly in UR when H_2_-supplementation was initiated but was greatly reduced after day 140 when the 48-h feeding regime was introduced (Fig. 6e). *Syntrophaceticus* is a well-known syntrophic acetate-oxidizing (SAO) bacterium that was discovered in a biogas reactor that relied on the energy from acetate oxidation to produce H_2_ and CO_2_ [16, 38]*.* SAO bacteria, which are syntrophic with hydrogenotrophic methanogens (*Methanothermobacter* in our case), can be inhibited by short or long-term H_2_ addition to their living atmosphere [21, 39]. Increased H_2_ partial pressure can inhibit SAO from a thermodynamic perspective because syntrophic sustainability is dependent on the H_2_/formate concentration, which is usually kept low by the methanogenic partners [44]. Interestingly, our study revealed that H_2_ addition at an H_2_:CO_2_ ratio of 2:1 promotes the growth of *Syntrophaceticus* while increasing the H_2_:CO_2_ ratio to 4:1 significantly reduces their abundance. In addition, the abundance of *Syntrophaceticus* of was maximum when the CW ratio was increased from 10 to 20%.

Similar to *Halocella*, *f_Hydrogenisporaceae_OTU_28*, was also affected by the increased stirring speed, seen as reduced abundance after 64 h in both reactors (Fig. 6f). *f_Hydrogenisporaceae_OTU_28,* a member of the *OPB54* class, have previously been reported to be involved in the fermentation of carbohydrates to produce acetate and H_2_ [45].

Our findings revealed that the H_2_:CO_2_ ratio, stirring speed, CM:CW ratio, and feeding frequency all had an effect on in situ BM, either on overall CH_4_ production or on CH_4_ production from H_2_ and CO_2_ conversion. However, it was only the H_2_:CO_2_ ratio and stirring speed that strongly affected the microbial community profile of the reactors.

## Conclusions

The current work demonstrates the feasibility of the hybrid biogas upgrading concept and identifies some challenges that must be tackled for future process improvement. When hybrid BM was used instead of in situ BM, it resulted in a 39% increase in CH_4_ yield. Furthermore, the hybrid BM setup resulted in a biogas containing 80% CH_4_ (excluding residual H_2_) and a total H_2_ utilization of 62%. The co-digestion of CM and AC aided in keeping the pH of the reactor below 8.1 (except at high stirring speed) during in situ BM. The addition of H_2_ at a H_2_:CO_2_ ratio of 2:1 resulted in stable operation of the in situ reactor system, while at higher ratio VFAs started to accumulate resulting in pH drop. The microbial analysis revealed that *Methanothermobacter*, a hydrogenotrophic methanogen, dominates both the control and the H_2_ reactors, with a higher abundance in the H_2_ reactor. The main factors affecting the microbial community composition were H_2_ addition and stirrer speed. The findings of our study may be useful to other researchers or biogas plant operators in developing processes for enhancing BM performance and methane yields. However, using electricity to produce H_2_ for biogas upgrading is probably only economically feasible in the case of an excess of renewable electricity at a low price.

## Materials and methods

### Inoculum and substrate

Thermophilic inoculum was obtained from two 10-L CSTRs digesting cow manure (CM) collected from a cow farm in Ås, Norway. Both reactors were operated at 55 °C and 20 days of hydraulic retention time. The same CM was also used as a model substrate for the present study. Increase in pH due to bicarbonate removal during in situ BM is commonly reported [12]. To limit pH increase during the in situ BM experiments, the CM was co-digested with acidic cheese waste acquired from the food pilot plant at Norwegian University of Life Sciences (NMBU). The cheese was produced only for experimental purposes [46] and discarded once the experiment was completed. The cheese waste (CW) was collected and was stored at 4 °C until further usage. Table 1 lists the characteristics of the inoculum and substrates used in this study.

### In situ BM setup

The setup comprised two 10-L CSTRs (control reactor, CR, and in situ upgrading reactor, UR), each with a 6-L working volume. The temperature of both reactors was maintained at thermophilic condition (55 °C). Three-blade Elephant Ear impeller operated in the down-pumping mode was used for mixing at 80 rpm. Approximately 300 g of substrate (90% CM: 10% CW) were fed into the reactors every 24 h after the same amount of effluent had been discharged. Initially, the organic loading rate was kept at 0.83 g_VS_ L^−1^ d^−1^. Starting day 64, H_2_ was injected into UR using a stainless-steel Mott sparger with a pore size of 2 µm, which was mounted at the bottom of the reactor. The sparger measured 12 cm in length and had a 12 mm outer diameter. The flow rate of H_2_ was initially set to 3 mL min^−1^ (H_2_:CO_2_ ratio  =  2:1). To increase the contact time between anaerobic microbes and H_2_, gas recirculation was introduced from day 64. A peristaltic pump was used to recirculate the output gas at gas recirculation rates of 7.63 mL min^−1^.

### Experimental parameters

In this study, various ways for optimizing gas–liquid mass transfer were investigated in order to increase the H_2_ consumption rate and CH_4_ content in biogas. The stirring speed was increased from 80 to 140 rpm, and the frequency of substrate feeding was reduced from once every 24 h to once every 48 h. Increased stirring speed in a CSTR improves gas liquid mass transfer and hence makes more H_2_ available for methanogens [1]. Moreover, it has been reported that reducing the frequency of substrate feeding may increase the abundance of hydrogenotrophic methanogens in a biogas reactor [28]. Thus, it was expected that a possible increased abundance of hydrogenotrophs due to less frequent substrate feeding would improve H_2_ uptake and CH_4_ formation.

The addition of H_2_ to the biogas reactor during in situ BM results in a significant increase in the H_2_ partial pressure. Some anaerobic bacteria are inhibited by high partial pressure, typically resulting in VFA buildup [1]. Thus, the H_2_:CO_2_ ratio was manipulated between 2:1 and 4:1 to investigate the optimal levels of H_2_ addition. Additionally, a pH increase to more than 8.3 has previously been seen as a result of bicarbonate removal, which can potentially cause inhibition [12]. To reduce the risk of pH rise, low pH cheese waste was co-digested with cow manure at different ratios (10 and 20%).

The experiment was divided into 6 different phases (I–VI) and Table 2 provides an overview of the corresponding parameter-settings. Stirring speed (80 vs 140 rpm), CM:CW ratio (90%:10% vs. 80%:20%), feeding frequency (24 h vs. 48 h), and H_2_:CO_2_ ratio (2:1 vs. 4:1) were varied from day 79–172 to examine how these factors influenced the process performance of the two reactors. The stirring speed was chosen based on our previous research [25], which found that 140 rpm was the optimum stirring speed for BM. Initially, a 2:1 H_2_:CO_2_ ratio was introduced into the reactor to avoid stressing the microbiome due to increased H_2_ partial pressure [21].

### Hybrid BM setup

A hybrid BM setup where the in situ reactor (UR) was combined with ex situ reactor (HR) was tested at the end of the experiment (day 173–203). The CR was not included in this experiment. The ex situ upgrading reactor was established using a 2-L bottle filled with 800 mL filtered and degassed inoculum (digestate from UR) and 108 g polyethylene packing materials with a surface area of 955 m^2^/m^3^ (Hel-X biocarriers, HXF13KLL + , Christian Stöhr GmbH & Co., Marktrodach, Germany). The inoculum from UR contained enriched cultures of hydrogenotrophic methanogens as a result of the addition of H_2_. The packing materials were submerged in HR for a week before hybrid BM experiment as a step to attach the biofilm to the packing materials. HR was kept at 55 °C. Once a week, 50 mL of the filtered and pasteurized CM was added to HR (nutrient supply) after the same amount of effluent had been discharged. All the biogas was transferred from the UR to the HR using a peristaltic pump and injected at the bottom through a diffuser. Figure 1a, b depicts the in situ and hybrid configurations.

### Sample analysis

Gas chromatography (GC) (SRI 8160C) with a flame ionization detector and N_2_ as the carrier gas was used to measure the gas composition (CH_4_, CO_2_, and H_2_). A standard biogas mixture (64% CH_4_ and 36% CO_2_) and a 10% H_2_ gas mixture (with 90% N_2_) (AGA Norway) were used for GC calibration on a regular basis. A digital pH meter (Thermo Scientific Orion Dual Star, USA) was used to measure pH of the digestate. pH measurement was performed immediately after the digestate was discharged from the reactors to avoid CO_2_ removal from liquid phase.

Digestates from the reactors were collected regularly for total solid (TS), volatile solid (VS), TAN and VFA analysis. TS, VS and TAN were measured according to the Standard Methods for Examination of Water and Wastewater (APHA, 2005). VFA samples were prepared following [25]. VFA concentration was determined using a high performance liquid chromatography (Dionex, Sunnyvale, CA, USA) with Aminex column as described previously [25].

## Microbial analysis

### DNA sampling and extraction

The liquid effluent from each reactor was collected regularly and stored at − 80 °C until DNA analysis. DNA extraction and sequencing were performed by DNASense (Aalborg, Denmark). The template DNA was extracted using the FastDNA Spin kit for Soil (MP Biomedicals, USA). The DNA extraction was performed following the manufacturer protocol except that samples were subjected to bead beating at 6 m/s for 4  ×  40 s [47]. DNA quantity and quality were assessed using gel electrophoresis with Tapestation 2200 and Genomic DNA screentapes (Agilent, USA). The Qubit dsDNA HS/BR Assay kit was used to determine the concentration of DNA (Thermo Fisher Scientific, USA).

### Sequencing analysis

Microbial community profiles were determined using 16S rRNA gene variable region V4 with primers [515FB] GTGYCAGCMGCCGCGGTAA and [806RB] GGACTACNVGGGTWTCTAAT [48]. The 25 µL PCR reactions contained (12.5 μL) PCRBIO Ultra mix, 400 nM primers and up to 10 ng of extracted DNA. The PCR thermal cycling consisted of a hot start step at 95 °C for 2 min, followed by 30 cycles of 95 °C for 15 s, 55 °C for 15 s, 72 °C for 50 s, and then a final 72 °C extension step for 5 min. For each sample, duplicate PCR reactions were performed, and the duplicates were pooled following PCR. The obtained amplicon libraries were purified using the standard protocol for CleanPCR SPRI beads (CleanNA, NL) with a bead to sample ratio of 4:5. The DNA concentration was quantified using Qubit dsDNA HS Assay kit (Thermo Fisher Scientific, USA) and the quality was confirmed by gel electrophoresis using Tapestation 2200 and D1000/High sensitivity D1000 screentapes (Agilent, USA). The purified libraries were pooled in equimolar concentrations and spiked with  >  10% PhiX control. The denatured library was sequenced on a MiSeq (Illumina, USA) using the Miseq Reagent kit V3.

### Bioinformatics

The sequenced amplicon libraries were trimmed for quality using trimmomatic v. 0.32 and merged [49, 50]. The reads were dereplicated and formatted for in the UPARSE workflow [51].Taxonomy was assigned using the RDP classifier as implemented in the script in QIIME and the SILVA database [52–54]. Bioinformatic processing was conducted by RStudio IDE (1.2.1335) (version 4.0.2) [47, 55, 56].

## Data Availability

The nucleotide sequence dataset used this study is available in the European Nucleotide Archive ENA (https://www.ebi.ac.uk/ena/browser/home) under project accession PRJEB46103.

## Abbreviations

AA: Acetic acid
BM: Biological methanation
CM: Cow manure
CR: Control reactor
CSTR: Continuous stirred tank reactor
CW: Cheese waste
GC: Gas chromatography
HR: Ex situ upgrading reactor
PA: Propionic acid
PtM: Power-to-methane
TAN: Total ammonium nitrogen
TM: Thermochemical methanation
TS: Total solid
UR: In situ upgrading reactor
VFA: Volatile fatty acids
VS: Volatile solid
